# The Transformation of Higher Education After the COVID Disruption: Emerging Challenges in an Online Learning Scenario

**DOI:** 10.3389/fpsyg.2021.616059

**Published:** 2021-02-11

**Authors:** Víctor J. García-Morales, Aurora Garrido-Moreno, Rodrigo Martín-Rojas

**Affiliations:** ^1^Faculty of Economics and Business, University of Granada, Campus Universitario de Cartuja, Granada, Spain; ^2^Faculty of Social Studies and Social Work, University of Malaga, Campus Teatinos (Ampliación), Málaga, Spain

**Keywords:** higher education, innovation, COVID-19, digital transformation, online learning

## Abstract

Crisis requires society to renew itself, albeit in a disruptive way. The current Covid-19 pandemic is transforming ways of working, living, and relating to each other on a global level, suddenly and dramatically. This paper focuses on the field of education to show how higher education institutions are undergoing radical transformations driven by the need to digitalize education and training processes in record time with academics who lack innate technological capabilities for online teaching. The university system must strive to overcome this situation to be competitive and provide high-quality education in a scenario of digital transformation, disruptive technological innovations, and accelerated change. To achieve these goals, this paper explains some barriers and challenges that universities encounter, as well as technological resources and methodologies they have used in the current scenario to transform higher education to face Covid-19 disruption. The discussion and conclusion synthesize significant insights that can be applied to the digitalization of education in the foreseeable future.

## Introduction

The disruption caused by the current Covid-19 pandemic is unprecedented, and the resulting economic and social measures have brought massive change (Krishnamurthy, 2020). To mitigate the spread of the virus, governments around the world have imposed social distancing measures, lockdowns, and cessation of personal contact outside immediate households. The pandemic is thus having a massive impact on educational activity. In a matter of weeks, entire education systems from elementary to higher education had to completely transform activity to evolve to an online teaching-learning scenario (Mishra et al., 2020). According to UNESCO, higher education institutions (HEIs) were closed completely in 185 countries in April 2020, affecting more than 1,000 million learners around the globe (Marinoni et al., 2020).

The reality of the new normal, disrupted by COVID effects, has involved a radical transformation of education and training, and one of the sectors undergoing dramatic digital transformation is global higher education (Dwivedi et al., 2020). The sudden forced closure of face-to-face teaching has led academics and students into “unfamiliar terrain” due to the need to adapt swiftly to total e-learning settings (Carolan et al., 2020). This sudden change has required universities to evolve toward online teaching in record time, implementing and adapting the technological resources available and involving professors and researchers who lack innate technological capacities for online teaching. The university system must be able to provide quality education in a scenario of digital transformation, disruptive technological innovation, and accelerated change in the educational framework. The emergence of disruptive innovation is a time of risk and uncertainty, but it is also a time of opportunities, bringing talent and innovation to the education system.

By definition, a disruption implies a sudden break or interruption. When applied to education, disruption involves a break from traditional, established educational models of knowledge transmission (Carolan et al., 2020; Mishra et al., 2020). Innovations that change the direction of education replace or displace existing models. They interrupt the functioning of established educational models in unexpected ways, first improving the model and then affording new ways of understanding its ongoing development. Disruptive educational innovation replaces existing methodologies and modes of knowledge transmission by opening new alternatives for learning. It also introduces new advances in education systems through information and communication technologies. This educational disruption considers both the student and the professor as engines of learning to promote an open curriculum enabled by new digital education. It also involves innovation in teaching methods; such as the development of new learning materials, mechanisms, and spaces; and the transformation of the role of students and the way they absorb and use educational knowledge. Disruptive innovations meet the needs of existing customers as well as the needs of currently available services (Christensen et al., 2006). Successful educational innovation and transformation must, however, be based on sustainability, scope, and scale (Carolan et al., 2020). The successful transformation of universities from old learning systems should foster a participatory culture, engage participants, and promote evidence-based decision making and transparent assessment of outcomes.

The new normal created by Covid disruption has accelerated the move toward online teaching. The current scenario has involved a rapid pedagogical shift from traditional to online class sessions, personal to virtual instruction, and seminars to webinars (Mishra et al., 2020). The impact of the pandemic will bring an era of radical technological transformation, with accelerated digitalization to the worldwide higher education system (Krishnamurthy, 2020). As universities must seriously rethink and redesign their educational offerings to face this new situation, Covid-19's disruptive effects have created not only fertile opportunities for transforming HEIs but also difficulties and challenges in this process (Carolan et al., 2020).

After presenting the gaps, we will attempt to fill them by shedding light on how HEIs are radically transforming education and training, evolving to digitalization in an extremely short time. To achieve successful transformation, universities should be aware of potential barriers and recognize new tools and systems, integrating this technology into the teaching-learning process. This paper will examine some significant technological resources and methodologies that universities are using, while also discussing the main obstacles and barriers encountered both by academics and students and at an institutional level. This article's novel contribution lies in its gathering of most articles on the topic of Covid-19 in HEIs to review the most common difficulties they identify and the solutions proposed to them by different countries globally.

## Transformation of Higher Education To Face COVID-19 Disruption

### Technological Resources and Methodologies Used

As a direct consequence of the social distancing efforts imposed by Covid and to maintain service during times of emergency, universities have experienced a large-scale transition to online learning (Krishnamurthy, 2020). In a short period of time, academics around the world have had to convert materials and methods rapidly to a format that is suitable for online delivery (Dwivedi et al., 2020). This transformation was hasty and compelled by circumstances. The pandemic forced a period of global experimentation with remote teaching (Govindarajan and Srivastava, 2020). Some studies refer to this new system as “emergency online education” (Marinoni et al., 2020). The system posed unprecedented challenges for students, who needed technical assistance, but also for staff and university leaders, who had to reinvent themselves in record time to keep campus operations running.

Although the process of digital transformation in higher education began years ago, the pandemic has accelerated it, leading to fundamental changes in a question of weeks. This technological transformation of education involves profound changes in teaching methodologies, essential competencies, and assessment methods, as most HEIs recognize (Jensen, 2019). In a virtual scenario, universities must evolve from a mostly “lecture-based learning” system toward “problem-based learning” methodologies, that engage students more actively (Marinoni et al., 2020). This transition from “in-person” to virtual education will have significant implications for the entire learning process, not only extensively modifying methods for assessing learning outcomes but also requiring reconsideration of the skills and competencies required of students in this new setting (Jensen, 2019).

As current social distancing measures will last for some time, education institutions must thoroughly redesign their service to face the new environment. To construct a well-designed online learning experience, universities should develop digital learning methodologies and provide digital learning contexts, tools, and support systems (Krishnamurthy, 2020).

Digital education requires appropriate infrastructure and technological platforms (e.g., Blackboard, Moodle, Microsoft teams), solid servers that can sustain the virtual workload, and methodological training of professors and students for online delivery using all the technical and educational resources available. Numerous webinars and guides are available for professors, and most universities have signed contracts with companies such as Microsoft that provide Office or Teams resources or technological platforms to strengthen virtual communication. At a global level, a wide variety of online communication platforms and solutions are available to help digitalize the entire teaching-learning process in the Covid-19 scenario (Mishra et al., 2020). In a recent empirical study conducted in a university context, these authors observed that the technologies most used to support teaching during the lockdown period were the university web platform; instant messaging tools (WhatsApp, Telegram); video-conferencing tools (Zoom, Skype, Google Hangouts, Google Meet); and educational apps (Google Classroom); combined with email and telephone conversations to maintain individualized contact with students. Other technologies were also generally useful (Cisco WebEx, GoToMeeting, Microsoft Teams, Monosnap, Loom, OBS).

The technological resources available provide multiple options for teaching, such as giving lectures by videoconference, sharing material (e.g., slides, videos, presentations), interacting through chats, creating debate forums or workgroups, supervising practical activities, evaluating and tutoring students, recording explanations and making them available to students, etc. Furthermore, these tools can be used synchronously or asynchronously and integrated. All of these resources must be supported, however, by an educational methodology to maintain students' attention and keep them involved in the course. To ensure clarity of the educational objective of each activity, instructors must design the audiovisual material, plan students' work time, and use the right tools for each activity—for example, for tutoring, videoconferencing activities, or student assessment. It is important to make sessions dynamic by introducing collaborative and formative tools. It thus also seems essential to introduce active methodologies for the interaction of students and professors, and that engage students in peer collaboration.

Various methodologies for online teaching and evaluation have emerged and proven useful in the current pandemic (the authors used some of these in remote teaching). The assessment process is very important, as it represents the culmination of the entire learning process. Table 1 provides a summary description of some of the main online assessment strategies and supporting digital technologies available. In addition to learning assessment, this article addresses other issues that should be borne in mind. Table 2 includes the main difficulties and breakthroughs different countries have encountered in the teaching-learning process during lockdowns, as they have made the massive migration or shift from traditional in-class face-to-face education to online education.

**Table 1:** Various resources/methodologies for student assessment in online teaching.

**Assessment methodologies**	**Description**	**Supportive technologies**
Diagnostic evaluation	Exercises, questionnaires, or tests that assess students' preconceptions, competences, information, etc., regarding the new topic	• Concept map • Questionnaires on Web platform • Online questionnaires • Interactive and gamified presentations
Evaluations using video tags	Students answer different questions by adding tags to a YouTube video. The professor can review students' answers by examining the labels. This process can be performed in groups or individually (as individual tests where students do not share their annotations)	• Videos on YouTube or published on the Web platform • Video annotations • Questionnaires on Web platform • Online questionnaires
Group and collaborative analysis	When not all exercises can be evaluated due to large number of students, one or more can work together. Evaluation may be anonymous or voluntary, and sequenced so all students are evaluated. At the time of evaluation, the exercises are shared so that the students better understand the quality criteria and their application to specific cases	• Videoconference platforms • Text and video annotations
Self-assessments	The student analyzes the work presented and evaluates it	• Online questionnaires • Rubrics • Questionnaire on Web platform
Co-evaluation or peer evaluation	Students evaluate the work of classmates in the group (intergroup) or work team (intragroup)	• Online questionnaires • Rubrics • Workshops on Web platform
360° evaluation	Contrasts evaluations of an individual or team exercise or tasks from different points of view: professor (hetero-evaluation) and/or students (co-evaluation or peer evaluation and self-evaluation)	• All tools available on Web platform that allow sharing of this evaluation, e.g., chat, digital rubrics, etc.
Objective tests	Exercises where students must select the correct answer or explanation to a problem from among several options	• Multiple response questionnaires on Web platform
Interviews	Interviews allow individual or group monitoring of a topic or topics, and can be considered as a continuous or final diagnostic evaluation	• Videoconference platforms
Ipsative assessment	Assessment that measures different moments of the process to assess progress. Students can observe their progress and achievements through repeated exercises and graphical representation of their evaluations	• Rubrics • Tools on Web platform
Oral partial or final exams	Review of learning achievement at the end of a process. Enables validation of learning achieved during the process Final or partial exams (need weighting) administered to students and graded or evaluated by the professor (hetero-evaluation) and the other students (co-evaluation)	• Online presentations • Videoconference platforms • Self-recorded videos by the student • Tests, reports, etc. included in tasks, plus anti-plagiarism tool on Web platform
Final evaluation	Tests that students must take	• Tests, reports, etc. that professors can publish on Web platform and that can be combined with the anti-plagiarism tool and resolved through Videoconference platforms

**Table 2:** Difficulties and breakthroughs in online learning-teaching.

**Authors**	**Difficulties encountered in massive “migration” from traditional in-class face-to-face education to online education**	**Breakthroughs**	**Country analyzed**
Aguilera-Hermida (2020)	• Situational and environmental challenges • Online educational challenges • Emotional challenges	• Technology Acceptance models: Attitudes, affect, and motivationPerceived behavioral controlCognitive engagement • Family time • New activities	• United States.
Bao (2020)	• Ambiguous future career goals • Lack of active academic involvement • More time spent in in-class study than in out-of-class study depending on students' study time	• 5 principles: Appropriate relevanceEffective deliverySufficient supportHigh-quality participationContingency plan preparation	• China.
Carolan et al. (2020)	• Prevailing institutional attitudes toward e-learning and pedagogy • Existing IT infrastructure • Availability of learning technology support • Staff digital literacy • Redeployment of academics	• Participatory culture • Distributed leadership • Engaged participants, shared and evidence-based decision-making • Transparent assessment of outcomes	• United States, • United Kingdom, • Australia.
European University Association (2020)	• Absence of economic and budgetary implications for higher education • Exacerbation of socially vulnerable stakeholders • Disguised learning and teaching practices • Internationalization programs • Learning difficulties for students • Socially disadvantaged students • Student stress	• Fostering of international mobility and cooperation • Major European networks and associations • Projects and mobility implementation • Stakeholder collaboration	• Europe.
Govindarajan and Srivastava (2020)	• Hybrid model?	• Face-to-face courses: Educational support on the ground: Instructional designers, trainers, and coaches to ensure student learning and course completionWhich students will remain on campus? • Online courses: Anonymized discussions about complementary technology issues, course design, course delivery, and evaluation methods	• United States.
Krishnamurthy (2020)	• Mental health of students • Mental health of employees • Short-term unbudgeted financial costs • Accelerated rates of student attrition and physical health of employees	• Transformation of university: • Technology Acceptance Model • Unbundle and re-invent teaching, learning, assessment, and certification • Focus on value, not just quality • Change in use and roles of faculty, mentors, and peer-to-peer learning • Transform business model	• United States.
Mishra et al. (2020)	• Time-bound online teaching-learning: Unstable network connection	• Use of e-teaching-learning tools available, such as Zoom, Google Meet, Facebook, and YouTube streaming • Use of Social Media, such as WhatsApp	• India.

*Source: The authors*.

### Emerging Barriers and Challenges in the Current Scenario

Covid-19's disruptive impact led to a rapid transformation of educational activity. As explained above, the rapid suspension of face-to-face teaching forced both students and professors to adapt to a wholesale shift in the teaching-learning process (Carolan et al., 2020). This adaptation was not obstacle-free, and some barriers and challenges emerged in this process (Marinoni et al., 2020; Mishra et al., 2020). To enable safe transition and achieve a successful transformation, universities must be aware of these potential obstacles and establish appropriate mechanisms to overcome them. Drawing on specific studies, we describe these barriers from the perspective of the main agents involved in the learning process: students, professors, and institutions (universities).

Students report that the major challenge in adapting to online learning was technical problems (Mishra et al., 2020). Some authors highlight the ways online education can amplify the digital divide (Govindarajan and Srivastava, 2020). To mitigate this barrier, institutions should mobilize resources to ensure that all students have access to a proper IT infrastructure and bandwidth connection, as well as specific support to solve technical problems (Carolan et al., 2020). To ensure an equitable student experience in this new scenario, universities must guarantee that students from less privileged socioeconomic backgrounds are not disadvantaged. Students also found it difficult to maintain attention in a purely online context, reporting the following significant barriers (among others) (Liang et al., 2020; Mishra et al., 2020): boredom, sense of isolation, lack of time to follow the different subjects, and lack of self-organizing capabilities. Professors also noted that isolation was a significant problem in designing the courses, indicating the need to find the optimum balance of individual student-centered learning and collaborative learning, fostering virtual communities of practice to enhance student peer engagement and collaboration (Carolan et al., 2020).

From the professors' perspective, this forced transformation was also stressful, as professors had to adapt quickly to new online techniques, with little or no training in some cases and in record time (Dwivedi et al., 2020). The sudden transition from face-to-face to distance teaching also required a teaching staff with diverse levels of readiness to use different pedagogies with specific competencies (Marinoni et al., 2020). The digital divide can also be applied to academics. Not all faculty members are comfortable in an online setting, and a generational divide may separate those who have relied on classical methods and never used technology tools from the younger faculty who may be more adept with newer technologies (Govindarajan and Srivastava, 2020). The main difficulties professors highlighted were the high demand for specific skills such as proficient computer knowledge, specific communication abilities for an online setting, proper handling of various teaching-learning tools, and the need to solve specific problems quickly during learning sessions. After an initial period of adaptation-experimentation to convert rapidly to remote teaching, however, academics highlighted some interesting lessons for overcoming barriers (Dwivedi et al., 2020). First, instructors should create an appropriate physical setting for online teaching, including lighting and sound. The specific content of class sessions should be thoroughly redesigned to adjust timing to online delivery and introduce group activities to motivate and engage students and encourage collaborative learning. As most universities will opt for a hybrid system in the near future that combines small face-to-face groups with online sessions, the challenge for academics will be to ensure that students in both situations experience high-quality learning (Dwivedi et al., 2020).

At the institutional level in universities, the move to emergency remote teaching in the Covid-19 pandemic involved a total disruption of business as usual (Krishnamurthy, 2020). To move toward a sustainable model for online learning, universities should use technology to re-invent teaching processes, transform assessment activities, change the use and roles of traditional Faculties and Schools (providing specific training), and focus on value through the reinvention and self-renewal of the service model. Promoting this digital transformation requires the cultivation of participatory culture, and students, professors, and administrators must work together to support and examine the changes implemented (Carolan et al., 2020). Universities also face additional barriers to this transformation, including financial constraints and the limits imposed by the current IT infrastructure (Krishnamurthy, 2020). Public universities will have to deal with diminishing budgets due to reduced government funds, and universities are experiencing a decrease in student enrollment due to the current uncertain economic situation. The IT-infrastructure available to universities will also limit opportunities to embrace full digital transformation, and some investments will be needed to enhance these technical capabilities. Despite all of these challenges, universities are quite positive about this transformation. In a recent survey conducted of institutions in all countries in the European Higher Education Area, most universities have confirmed that they have plans to explore new ways of teaching (92%) and enhance digital capacity (75%) beyond the crisis (European University Association, 2020).

We conclude this section by drawing on recent literature and a proactive approach to summarize some key insights for higher education's transformation toward online education. First, institutions need to improve their technological infrastructures, while at the same time ensuring that all students have equal access to the technological resources needed. This step requires a financial investment to enable a real digital transformation (Jensen, 2019). Another major obstacle to technological transformation is the human factor. There is a strong need for institutional leadership and support, involving the different stakeholders (faculty, students, technical staff) in the change process. The successful transformation of higher education requires faculty development and specific policies to improve crisis management readiness and increase institutional resilience to address new challenges in the near future (Marinoni et al., 2020). Finally, the increase in digitalization and available information leads to new ethical questions regarding online security and rights to data privacy. Universities must also address these issues by developing codes of conduct to ensure transparency and create a safe, trustworthy environment for online learning (Jensen, 2019).

## Discussion and Conclusion

The disruptive impact of Covid-19 and the availability of digital technologies that can support online learning present an unprecedented opportunity for the transformation of higher education at a global level. We are all involved in a digital world, and the phenomenon of online learning is here to stay. After some months of online experiences, a paradigm shift has occurred in university education. Online teaching has gained relevance and ensured its continuance even after the Covid-19 pandemic. Our examination reveals the use of a plethora of technological tools and platforms to support online learning: web-based learning platforms, video-conferencing tools, Massive Open Online Courses (MOOCs), streaming conferences, instant messaging tools, and educational apps, among others, to support new methodologies to enable learning processes. As this transition to online learning was hasty and forced by circumstances, however, the various actors in the learning processes (students, professors, universities) encountered several barriers in adapting to this new setting. Universities must be aware of these barriers and mobilize resources to overcome them in the short term, paying special attention to the digitalization of learning processes and offering specific technical training to professors, administrative staff, and students. We do not yet know what the shift to virtual learning will mean for the future of higher education at global level, but it is clear in the current scenario that universities should develop a sophisticated combination of face-to-face and online learning to harness the potential of the technological tools available to meet students' expectations and enhance their learning experience in the current digital environment. The main contribution of this paper is thus to observe online teaching from different perspectives, with a primary focus on connectivism (Millwood, 2011), based on Bandura's theory of constructivism, while taking into account both assessment problems and the main difficulties in online teaching and learning caused by Sars-Covid-2 outbreaks throughout the world.

## Author Contributions

All authors contributed equally to the manuscript and approved the version submitted.

## Conflict of Interest

The authors declare that the research was conducted in the absence of any commercial or financial relationships that could be construed as a potential conflict of interest.
